# Investigation of the acid/base behaviour of the opium alkaloid thebaine in LC-ESI-MS mobile phase by NMR spectroscopy

**DOI:** 10.1098/rsos.170715

**Published:** 2017-10-04

**Authors:** Michelle G. Carlin, John R. Dean, Jonathan L. Bookham, Justin J. B. Perry

**Affiliations:** Department of Applied Sciences, Northumbria University, Ellison Building, Newcastle upon Tyne NE1 8ST, UK

**Keywords:** opium alkaloids, thebaine, LC-ESI-MS, NMR, poppy seeds

## Abstract

As part of a research programme to establish an analytical method for the simultaneous detection of the five major opium alkaloids in poppy seeds by liquid chromatography–electrospray ionization–mass spectrometry (LC-ESI-MS) it was discovered that the inclusion of thebaine produced two peaks for the same compound. This was in contrast to the effective simultaneous detection, by LC-ESI-MS, of morphine, codeine, papaverine and noscapine. The presence of these two peaks for thebaine was investigated using nuclear magnetic resonance spectroscopy with deuterated solvents to emulate the mobile phase conditions experienced. It was found that the presence of 80%, or higher ratios of, water caused two epimeric forms of thebaine to be formed; this explained the presence of two peaks on the chromatogram. In contrast, when a lower water content was used with 1% acetic acid, one stable form of thebaine could be analysed and resulted in a single peak visible in the subsequent chromatography.

## 1. Introduction

*Papaver somniferum* L. (the opium poppy) is an annual crop cultivated worldwide but is legitimately grown by the pharmaceutical and food industries in France, Spain, Turkey, Holland, Hungary, Poland, Romania, Czech Republic, Slovakia, the former Yugoslavia, India, Central and South America, Canada, Australia and Iran (http://www.incb.org/incb/en/publications/annual-reports/annual-report-supplement-2015.html). The world's illicit crop of opium originates predominantly in an area well known for the production of opium and heroin known as the ‘Golden Triangle’ (Myanmar, Lao People's Democratic Republic and Thailand) and Afghanistan. These areas are also well known for drug smuggling (https://www.unodc.org/wdr2016/en/opiates.html).

*Papaver somniferum* L. is cultivated for the pharmaceutical industry as a source of morphine, codeine, thebaine and oripavine as narcotic raw materials (NRM). The NRM are extracted from poppy straw from which the pharmaceutical industry synthesizes active pharmaceutical ingredients such as morphine sulfate, codeine phosphate, hydrocodone and naloxone (http://www.incb.org/incb/en/publications/annual-reports/annual-report-supplement-2015.html). *Papaver somniferum* L. is an herbaceous plant that when grown in the Southern Hemisphere is generally sown in July, with the crop managed between August and December, and harvested between January and April. This final phase includes harvesting, assessment of crop quality and payment. In the Northern Hemisphere the crop is generally sown in late autumn/early winter [1]. A by-product of the process of harvesting poppy straw is poppy seeds (http://www.incb.org/incb/en/publications/annual-reports/annual-report-supplement-2015.html). This source of poppy seeds is used by the food industry and these seeds are included in cakes, on bread products and sold to supermarkets and specialist shops for use in cooking/baking recipes.

It was initially thought that the seeds and any products derived from them would not contain any alkaloid compounds due to the fact that the seeds develop after the latex (http://www.incb.org/incb/en/publications/annual-reports/annual-report-supplement-2015.html). In the late 1970s, it was noted that poppy seeds contained alkaloids found in opium [2]. However it was not until the 1980s that research in this field escalated and in 1998 a paper was published by Meadway *et al.* which highlighted that it was possible to fail a urine drug test after the consumption of a bread product containing poppy seeds [3]. Over the last 10–15 years it has become increasingly apparent that the presence of alkaloids in the food chain is a problem and can potentially lead to serious repercussions with respect to workplace and roadside drug testing [4–7]. Being able to differentiate between poppy seed consumption and other sources of morphine/codeine in biological material (e.g. heroin) would be highly advantageous in these circumstances.

**Figure 1.**
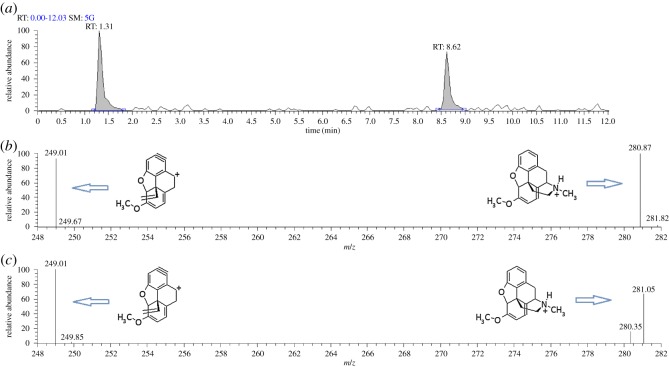
(*a*) Original chromatogram for thebaine showing two peaks, (*b*) mass spectrum for the peak at 1.31 min, and (*c*) mass spectrum for peak at 8.62 min.

**Figure 2.**
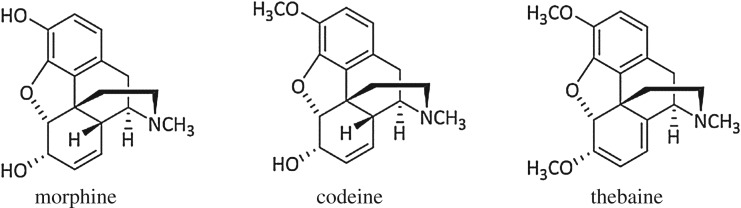
Chemical structures of three major opium alkaloids.

For this reason, a liquid chromatography–electrospray ionization–mass spectrometry (LC-ESI-MS) method was established which could detect morphine, codeine, thebaine, papaverine, noscapine and deuterated morphine as the internal standard. These compounds, with the exception of the deuterated internal standard, were chosen as they are the five major alkaloids present in *Papaver somniferum* L. They comprise the largest per cent, by weight, of dried opium and are therefore more likely to be identified in poppy seeds and poppy seed containing food products [8,9]. During the method development stage it was noted that all compounds produced one single peak in a chromatogram but that there were two peaks present in the chromatogram for thebaine with both having very similar mass spectral data (figure 1), even when selective ion monitoring and MS/MS was used. Due to the fact that the chemical structure of thebaine is very similar to morphine and codeine (figure 2) using the peak with the more similar retention time to morphine and codeine (approximately 1.3 min) was used to create a calibration curve. However, this did not provide a linear response nor did a calibration curve for the other signal at 8.62 min. This was in contract to the other four alkaloids which routinely produced linear responses with a coefficient of determination (*R*^2^) greater than 0.999.

The scientific literature in this field contains very few references to thebaine in LC-MS methods despite the evident need for a broad spectrum method of detection and quantitation of alkaloids in opium, poppy seeds or biological matrices. This might suggest that thebaine has a history of being problematic in chromatographic separations. In order to investigate whether structural effects pertaining to thebaine itself might be the cause of this anomalous behaviour, a series of experiments were carried out using nuclear magnetic resonance (NMR) spectroscopy to examine its behaviour in solution. Experiments were designed to replicate the changes in mobile phase composition encountered by the thebaine analyte during analysis by LC-ESI-MS. There is a long history of the use of NMR spectroscopy to elucidate the structure of alkaloids. In 1975, Terui *et al*. [10] reported the ^13^C spectra for morphine alkaloids thebaine and sinomenine. In 1984, Theuns *et al.* [11,12] analysed the structure of thebaine using both ^13^C and ^1^H NMR and most recently Caldwell *et al*. [13] reported the configurational analysis of thebaine and codeine using ^13^C and ^1^H NMR in 1996. In this article, we report experiments which were performed to analyse structural behaviour in a range of solvents which replicate the changes in mobile phase composition encountered by the thebaine analyte during analysis by LC-ESI-MS.

## 2. Experimental

### 2.1. LC-ESI-MS

LC-ESI-MS was performed using an LC Surveyor system hyphenated to an LCQ Advantage ion trap mass spectrometer (both instruments ThermoFinnigan, Hemel Hempsted, UK). A Gemini 3 µm C18, 100 × 2.00 mm column fitted with a Gemini C18, 4.0 × 2.0 mm guard column (Phenomenex, Cheshire, UK) was used and was thermostatically controlled at 30°C. The LC method employed two separate mobile phases: acetonitrile + 1% acetic acid and water + 5% acetonitrile + 1% acetic acid. LC-grade water was obtained from a Millipore purification system. The mass spectrometer was operated in positive electrospray ionization mode. Selection and tuning of the mass spectrometer settings for each of the alkaloid analytes, including thebaine, were performed using direct infusion, involving the direct introduction of each of the analytes dissolved in methanol. LC-MS data analysis was carried out using XCaliber 2.0 software package supplied with the Thermo Finnigan LC system and LCQ Advantage ion trap mass spectrometer. The LC conditions for the analysis are shown in table 1.

**Table 1.** Liquid chromatography conditions for the analysis of thebaine and other opium alkaloids.

*mobile phase composition* solvent A: acetonitrile + 1% acetic acid solvent B: water + 5% acetonitrile + 1% acetic acid			
time (minutes)	%A	%B	flow rate (μl min^−1^)
0.00	0	100	200
10.00	65	35	200
12.00	65	35	200
14.00	0	100	300
20.00	0	100	300

### 2.2. NMR spectroscopy

Thebaine, purchased from Sigma Aldrich (Poole, UK), was analysed using ^13^C and ^1^H NMR. ^1^H NMR spectra were obtained using a JEOL ECS 400 NMR spectrometer operating at a frequency of 400 MHz, using 8–32 scans, a relaxation delay of 5 s, and a flip angle of 45° (5 µs pulse). Spectra were Fourier transformed typically into 32 000 data points using standard exponential window with a line broadening factor of 0.2 Hz. ^13^C spectra were obtained at a frequency of 100.53 MHz, from 128–1048 scans, a relaxation delay of 2 s, and a flip angle of 30° (2.7 µs pulse). Spectra were Fourier transformed typically into 64 000 data points using standard exponential window with a line broadening factor of 0.5 Hz. Two-dimensional spectra (COSY, HSQC and HetCor) experiments were conducted using standard JEOL automated acquisition and processing parameters. Thebaine standards for analysis by NMR spectroscopy were prepared by dissolving approximately 10 mg of the powdered thebaine standard into a 1 cm^3^ volume of the relevant deuterated solvent or solvent mixture incorporating 1% of deutero-acetic acid (CD_3_COOD). Deuterated solvents were purchased from Goss Scientific (Crewe, UK).

## 3. Results and discussion

In the HPLC method described, two separate mobile phases were used: acetonitrile + 1% acetic acid and water + 5% acetonitrile + 1% acetic acid. It was decided to establish if there was any difference in the chemical structure of thebaine as detectable by NMR spectroscopy with (i) varying proportions of the solvent acetonitrile to aqueous component and (ii) varying amounts of acid in the solvent. It was proposed that the changes in pH and solvation may produce ionized and unionized forms of the thebaine with different enough structures to provide chromatography showing two peaks (figure 1). Solutions were prepared for ^1^H NMR in varying ratios of deuterated water (D_2_O): deuterated acetonitrile (CD_3_CN) + 1% deutero-acetic acid (CD_3_COOD) to investigate this effect.

**Figure 3.**
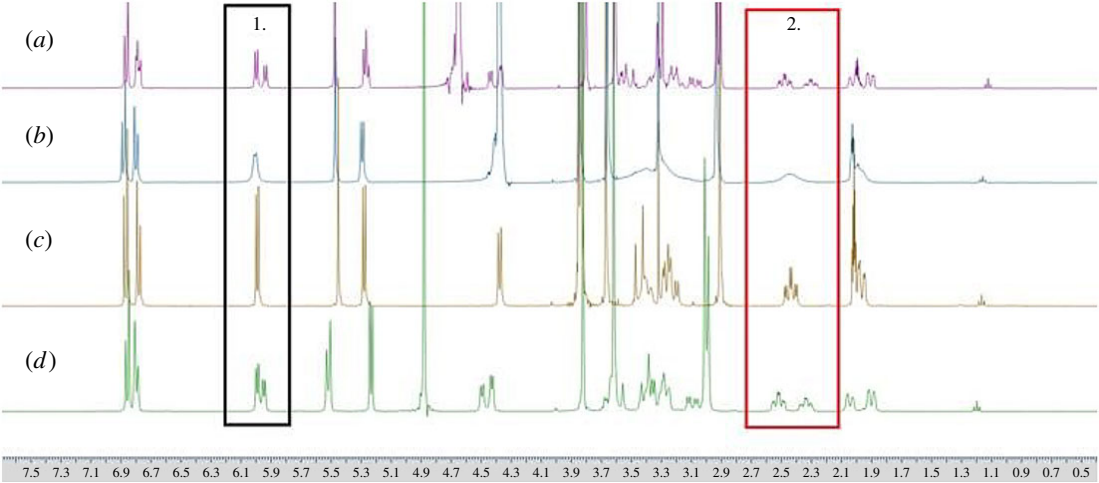
^1^H NMR spectra for thebaine in (*a*) D_2_O : CD_3_CN ratio 80 : 20 (v/v), (*b*) D_2_O : CD_3_CN ratio 50 : 50 (v/v), (*c*) D_2_O : CD_3_CN ratio 20 : 80 (v/v), (*d*) 100% D_2_O. All solutions contain 1% CD_3_COOD. *X*-axis units are ppm.

Thebaine (figure 2) contains 19 carbon and 15 hydrogen environments the signals from some of which are highly sensitive to solvent and acid content variation. Figure 3 shows the ^1^H NMR spectra for thebaine in four different solvent ratio mixtures of deuterated water and deuterated acetonitrile. The ^1^H NMR spectrum of thebaine in only D_2_O containing 1% CD_3_COOD is shown in figure 3*d*. At this concentration of acid, it is reasonable to assume the bridgehead nitrogen atom to be effectively fully deuterated and thus the potential for two structurally distinct but similar epimeric forms can be confidently postulated [14].

The presence of two epimeric forms (in an approximately 58 : 42 ratio as determined by integration) is evident on inspection of the ^1^H NMR spectrum, and is exemplified by pairs of signals for each proton environment in the thebaine structure. This signal pairing is most obvious for the hydrogen environments closer to the bridgehead nitrogen, for example the comparable (triplet of doublets) pair of signals at *ca* 2.4 and 2.2 ppm which each derive from one of the CH_2_-N protons on the bridgehead ethylene units of the two epimers (figure 3, box 2).

At more remote locations within the molecule, the chemical shift differences between the analogous H atoms on the epimers are less profound and the two separate signals cannot always be observed by simple inspection of the 1D ^1^H NMR. However, assignments of signals on NMR spectra were made in reference to previously published analyses [10–13] and use 2D HH and HC COSY to support these conclusions. The ^1^H NMR spectrum of thebaine in an 80 : 20 v/v D_2_O/CD_3_CN solvent mix containing 1% CD_3_COOD (figure 3*a*) indicates the presence of two epimeric forms in an approximate ratio of 58 : 42. In contrast, when the D_2_O/CD_3_CN ratio is reversed (to 20 : 80 v/v) a less complex spectrum (figure 3*c*) results without any evidence of the signal pairing at higher water proportions. This suggests either the presence of a single epimer form of the thebaine-D^+^ complex ion, or the presence of the two forms exchanging rapidly on the NMR timescale. At a 50 : 50 v/v solvent ratio (figure 3*b*), broad signals are obtained which are consistent with a rapid exchange between the two epimer forms and this therefore suggests that spectrum figure 3*c* is indeed that from two rapidly exchanging epimer forms; however, not as rapid as the exchange observed in figure 3*b*.

**Figure 4.**
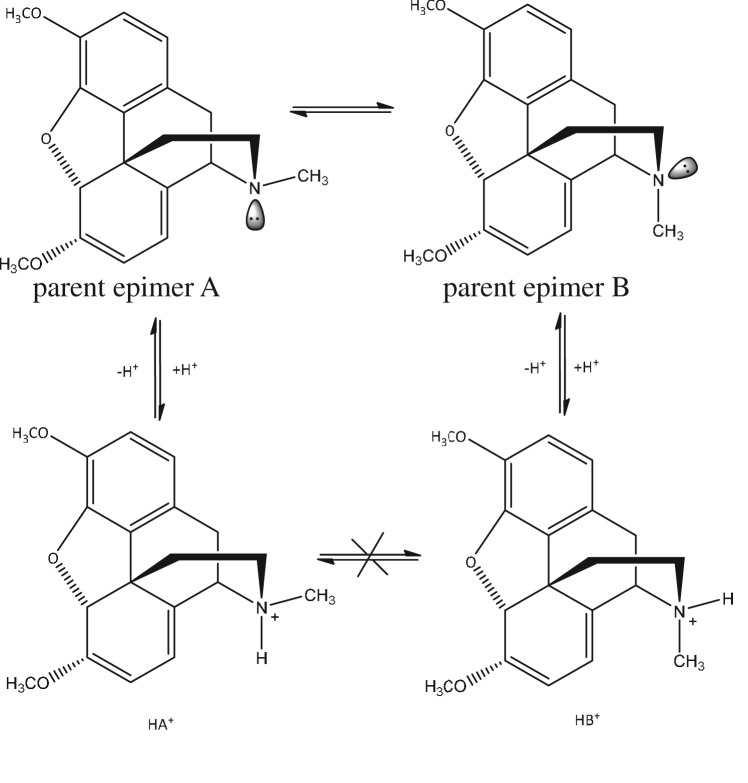
Formation of thebaine-D^+^ complex ions, HA^+^ and HB^+^, from epimers A and B*.*

The profound changes in the spectra across these solvent mix ratios suggest that the exchange rate is regulated primarily by the solvent composition (with water being the dominant apparent mediator), rather than the pH of the solvent, which is consistent with the findings of Caldwell *et al*. [13]. However, further studies we have undertaken on thebaine and other opiate alkaloids (morphine and codeine due to their similar chemical structures to that of thebaine) at lower acid levels reveal that, at these levels, the rate of exchange of epimer forms is indeed pH dependent and this suggests that proton exchange is a key process in the overall exchange mechanism under some solvent conditions. It appears that at higher concentrations of D_2_O, the nitrogen is saturated which may result in the two epimers. When the concentration of D_2_O is reduced, this in turn leads to a decrease in the D^+^ ion, leaving the nitrogen no longer saturated resulting in the exchange of epimers shown in figure 4. The ^1^H NMR spectra of the free-base form of thebaine in three standard but diverse NMR solvents (CDCl_3_, DMSO-d6, CD_3_CN without acid incorporated) are consistent with the presence of a single isomeric form in each case (the free base is not sufficiently soluble in D_2_O to state this for aqueous solutions), although the theoretically stereogenic nitrogen centre of thebaine also allows for epimer formation. However, it is reasonable to assume that the presence of the lone pair on the pyramidal nitrogen atom of the free base form facilitates rapid pyramidal inversion at the N atom such that epimer formation is transient and there is a rapid exchange between the epimer types. The aforementioned spectra of thebaine are therefore also consistent with that of a rapidly interconverting epimer pair. In the presence of acid (normally as H^+^ but as D^+^ in this NMR study) the N atom is quarternized to the corresponding ammonium salt which then is unable itself to undergo pyramidal inversion (figure 4), although as part of a wider study of thebaine and related species we have observed that at lower acid concentrations exchange between epimer forms is promoted, presumably mediated by a process of proton exchange (i.e. loss), inversion and reprotonation which is constrained at higher acid concentrations (unpublished results).

**Figure 5.**
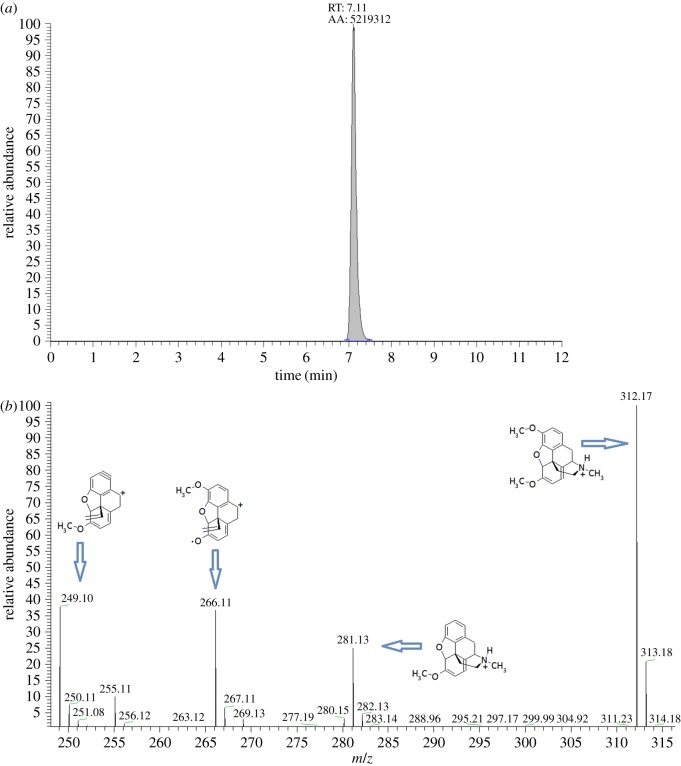
(*a*) Chromatogram for thebaine showing only one peak. (*b*) Extended mass spectrum showing the expected peaks at *m/z* of 281 and *m/z* 249, as well as one extra fragment at *m/z* 266 and the molecular ion, *m/z* 312. [15].

**Figure 6.**
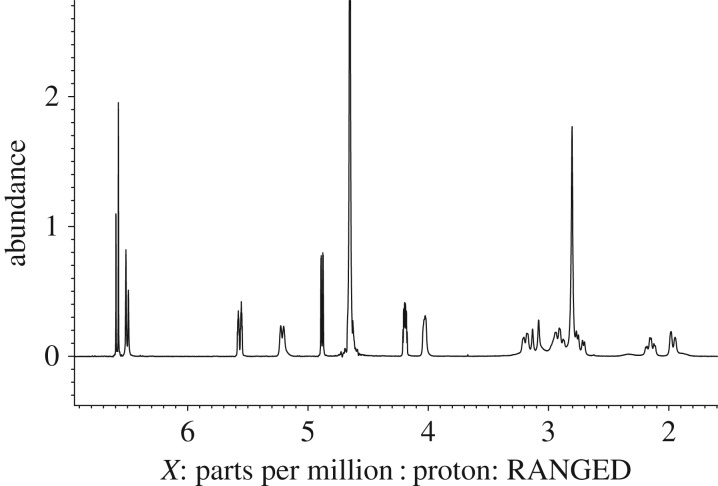
^1^H NMR spectrum of morphine in D_2_O.

In this NMR study, one structural form of thebaine was obtained by employing 20 : 80 v/v D_2_O/CD_3_CN solvent mix containing 1% CD_3_COOD. To transfer these conditions for HPLC analysis, 20 : 80 v/v H_2_O/CH_3_CN solvent mix containing 1% CH_3_COOH was used and a standard of thebaine was injected into LC-MS, with all other parameters kept constant. Only one peak was obtained for thebaine (figure 5) on the resulting chromatogram. This strongly suggests that the issue of two chromatographic peaks for thebaine was due to two epimeric forms and that this could be removed by manipulating solvent conditions to those suggested by NMR analysis. Similar work was carried out using morphine, since it is structurally similar to thebaine. Morphine was dissolved in D_2_O and analysed by NMR under the same conditions as thebaine, with the resulting spectrum in figure 6. Noticeable in this spectrum is the presence of fairly broad resonances (e.g. those at approximately 5.2 and approximately 4.0 and the groups around 2 and 3 ppm). At first glance, this spectrum appears to be from a single epimer although, in principle again there are two epimeric protonated forms of morphine (as with thebaine) but little literature on this appears evident. There are some ‘interesting’ shallow (weak) broad humps (e.g. those at 2.3 and 1.9 ppm) in the spectrum. Given that thebaine has two clearly distinguishable epimers in its NMR spectrum in D_2_O there is no reason why morphine should not show similar behaviour: these weak/broad signals may herald this. However, unlike thebaine, morphine shows only one peak in the resulting chromatogram when analysed under the same LC-MS conditions. Very similar results to morphine have been found for codeine.

## 4. Conclusion

NMR spectroscopy has been used to study an anomaly in the chromatographic analysis of thebaine. It was found that in solution, thebaine exhibits long-lived epimeric behaviour of its cationic form in a commonly used acidic mobile phase. It was found that the use of a H_2_O/CH_3_CN (20 : 80 v/v) ratio, as suggested by NMR spectroscopic investigation of solvent mixtures, provided a suitable mobile phase which proved ideal for thebaine analysis by LC-ESI-MS.

## Supplementary Material

NMR Spectra of Thebaine

## Data accessibility

Further data can be obtained in the electronic supplementary material and by contacting the corresponding author, e-mail: john.dean@northumbria.ac.uk, telephone: +44(0)191 227 3047.
