# Supplementary material for: Investigation of the acid/base behaviour of the opium alkaloid thebaine in LC-ESI-MS mobile phase by NMR spectroscopy
Source: R Soc Open Sci. 2017 Oct 4;4(10):170715. doi: 10.1098/rsos.170715 (PMC5666260; doi:10.1098/rsos.170715)
Supplement: NMR Spectra of Thebaine [file rsos170715supp1.docx]

**Investigation of the acid/base behavior of the opium alkaloid thebaine in LC-ESI-MS mobile phase by NMR spectroscopy**

Michelle G. Carlin, John R. Dean, Jonathan L. Bookham, Justin J.B. Perry

Department of Applied Sciences, Northumbria University, Ellison Building, Newcastle upon Tyne, NE1 8ST, UK

**Electronic Supplementary Material**

All spectra from JEOL ECS 400 NMR spectrometer operating at 399.8 MHz using Delta 5.03 software package. Simulations using gNMR software

**Experiment A: Initial spectrum**

~ 10 mg parent (free base) in CDCl_3_ (~1 cm^3^).

Upper plot: Experimental NMR spectrum.

Lower plot: Simulated NMR spectrum.

**Comments/observations:**

1. NMR analysis generally accords with that of Caldwell et al. (1996).^13^

**Experiment B: Acidification of Thebaine**

NMR sample in CDCl_3_ as above treated with excess (ca 1%) CD_3_COOD.

Below upper: ^1^H NMR spectrum of acidified CDCl_3_ sample

Below lower: Original spectrum (free base)

**Comments/observations**

1. Spectrum of protonated form is stable over time and on heating to 60°C.
2. Spectral ‘impurities’ include residual CHCl_3_ (~7.25 ppm), water (1.5 ppm lower plot) which is lost through ‘D_2_O’ style ‘shake’ in upper spectrum, residual CHD_2_COOD/H multiplet at ~ 2.0 ppm (upper plot). None of these impurities are chemically significant.
3. Spectral changes associated with protonation are mainly chemical shift changes and most pronounced for signals deriving from sites nearest the protonation centre, changes to coupling constants are small (see spectral simulation below (lower plot = simulated spectrum)

**Comments/observations (continued)**

1. Spectrum of acidified sample shows generally well resolved peaks although one or two signals are broader probably as a result of exchange processes and/or numerous small but unresolved couplings.
2. Spectrum appears at first sight to be of a single isomer (epimer) of the protonated form although the possibility of an exchange between epimers (that is approaching fast on the NMR timescale) is not excluded.

**Experiment C:** LC Solvent gradient effect mimicry.

Thebaine solutions (~10 mg) in (ca 1 cm^3^) solvent mix of D_2_O/CD_3_CN in various ratios incorporating a fixed 1% CD_3_COOD to mimic the range of solvent ratios in standard LC-MS chromatographic procedure for thebaine determination (Note: deuterated solvents used for NMR reasons). The presence of deutero- acetic acid protonates (deuterates) the thebaine N atom thus promoting solubility in the polar LC eluant system. NMR spectra are therefore (presumably) of D^+^ salts of thebaine rather than the parent free-base (the comparable NMR chemical shift patterns to those seen in Experiment B support this).

Three example spectra shown.

Top plot: D_2_O:CD_3_CN ratio 80:20 (v/v)

Middle plot: D_2_O:CD_3_CN ratio 50:50

Bottom plot: D_2_O:CD_3_CN ratio 20:80

**Key observations/comments:**

1. Spectra have been manually aligned to mitigate solvent effects on chemical shifts.
2. Residual water signal moves from ~3.85 ppm to ~4.4 ppm to ~4.7 ppm from bottom to top plot and increases in intensity reflecting the increasing aqueous nature of the solvent mix. Only in the middle spectrum does this signal obscure any thebaine signal.
3. Residual acetonitrile multiplet solvent signal at ~2 ppm diminishes bottom to top and partially obscures one thebaine signal throughout.
4. **Bottom spectrum** (predominantly acetonitrile) shows a clean spectrum of what appears to be a single D^+^ salt of thebaine. Alternatively the spectrum is consistent with a rapidly exchanging epimer pair (two isomeric/epimeric forms are possible depending on the stereochemistry of the chiral ammonium centre that results from quaternisation).
5. **Top spectrum** (predominantly D_2_O) shows the presence of two isomeric forms (ca 55:45 ratio) of the Thebaine-D^+^ complex ion with spectra consistent with that reported by Caldwell et al.^13^ and consistent with two epimeric forms in solution. The corresponding spectrum in 100% D_2_O/D^+^ has been fully simulated (shown below) as further confirmation .
6. **Middle Spectrum** (50:50 D_2_O : acetonitrile) shows broad signals consistent with a slow exchange (on the NMR timescale) of the two isomeric/epimeric forms of thebaine-D^+^ complex.

Top plot: Experimental spectrum of Thebaine in 100% D_2_O/D^+^)

Bottom plot: Simulated spectrum (ThebaineD^+^ x2 in D_2_O)).

**Some Conclusions**

The protonation of thebaine is fully realised at 1% acid concentration but the ratio and/or exchange rate of the epimer pair appear be significantly solvent dependent. In 100% aqueous (containing 1% D^+^) 2 epimeric forms, with similar but subtly different NMR spectra, are clearly visible and formed in an approximate 45:55 ratio. Again the greatest variation in parameters between forms are the chemical shifts of protons near the stereogenic nitrogen protonation centre. The broadness of many of the NMR signals is consistent with slow exchange between epimers although the spectra are resolved well enough for an essentially full NMR analysis and assignment. An initial VT NMR and exchange simulation / calculation exercise indicates an exchange rate of the order of 5 Hz at ambient temperature (19 °C) rising to ~30 Hz at 50 °C but this needs to be investigated with more precision. In 80:20 acetonitrile/water a NMR spectrum consistent either with a single isomer or with a rapidly exchanging isomer-pair system is observed. Thus, either the ratio of isomers appears to depend on water concentration (or acetonitrile concentration depending on your viewpoint) or the exchange rate between them is significantly solvent dependent, or possibly both. In 50:50 acetonitrile/water the NMR peaks are noticeably broadened and consistent with a fairly rapid exchange between isomers but it is not possible to establish the proportions of the exchanging forms in this solvent system. The formation/exchange process is fully reversible, that is, all spectra are reproducible via back/forward titration with either single solvent.

Next steps: Assuming exchange is via a protonation/deprotonation pathway rather than any non bond-breaking process the exchange rate should be dependent on acid concentration. This therefore allows a further study of the exchange in 50:50 acetonitrile/water at variable acid concentration – this would then establish whether the solvent proportions controls either isomer proportions or exchange alone, or both jointly.

**Experiment D.**

NMR spectra of (top to bottom) samples Thebaine 1-6 as prepared below showing the slowing of exchange at higher acid concentration. Approximate exchange rates determined by exchange simulation using gNMR are indicated in the table.

| **Thebaine** | **CD_3_COOD (% v/v)** | **D_2_O (% v/v)** | **CD_3_CN (% v/v)** | **Exchange Rate*(Hz)** |
| --- | --- | --- | --- | --- |
| 1 | 0.99 | 49.5 | 49.5 | 115 |
| 2 | 1.96 | 49.0 | 49.0 | 60 |
| 3 | 3.85 | 48.1 | 48.1 | 22 |
| 4 | 7.41 | 46.3 | 46.3 | 8 |
| 5 | 12.06 | 44.0 | 44.0 | 3.5 |
| 6 | 20.09 | 40.0 | 40.0 | 1.5 |

**Below:** Experimental (bottom) and simulated (top) partial (higher chemical shift region) NMR spectra of Thebaine 3 using an exchange rate of 22 Hz (presented back-to-back)

**Below** – overlay of line fitting of the simulated exchange (22 Hz) and experimental spectra for the exchange of specific epimeric signals at ~2.5 and 2.3 ppm in Thebaine 3.
